# Weight affects survival of primary total knee arthroplasty: study based on the Danish Knee Arthroplasty Register with 67,810 patients and a median follow-up time of 5 years

**DOI:** 10.1080/17453674.2018.1540091

**Published:** 2018-12-05

**Authors:** David Gøttsche, Kirill Gromov, Petra H Viborg, Elvira V Bräuner, Alma B Pedersen, Anders Troelsen

**Affiliations:** a Department of Orthopedic Surgery, Copenhagen University Hospital Hvidovre, Clinical Orthopaedic Research Hvidovre (CORH), Denmark;; b The Danish Clinical Registries (RKKP);; c Department of Growth and Reproduction, Rigshospitalet, University of Copenhagen, Copenhagen, Denmark;; d Department of Clinical Epidemiology, Aarhus University Hospital, Aarhus, Denmark

## Abstract

Background and purpose — Obesity is a rising issue worldwide and growing evidence supports poor outcome amongst obese patients following total knee arthroplasty (TKA). Using nationwide registries we investigated the association between bodyweight and risk of revision of primary TKA.

Patients and methods — All primary TKA performed during 1997–2015, weight at time of primary TKA and subsequent TKA revisions were identified in the Danish Knee Arthroplasty Register (DKR). Data on comorbidities and a priori selected confounding variables were collected from nationwide registries. The association between weight and 1st time TKA revision was calculated as both crude and adjusted hazard ratios (aHR) with 95% confidence intervals (CI) using Cox regression.

Results — Of 67,810 identified primary TKAs, 4.8% were revised within a median follow-up time of 5.4 years. No association between weight and risk of any revision in patients aged 18–54 and 55–70 years was found. Increased risk of any revision was seen in patients >70 years, 80–89 kg (aHR =1.5, CI 1.2–1.8), 90–99 kg (aHR =1.7, CI 1.3–2.1) and patients >99 kg (aHR =1.6, CI 1.3–2.1), as well as those weighing 45–60 kg (aHR =1.4, CI 1.1–1.9) compared with same aged patients weighing 70–79 kg.

Interpretation — We found a complex association between weight and knee arthroplasty survival. There was an increased risk of any revision in patients older than 70 years of age weighing <60 kg and >80 kg. Patients aged 18–55 years weighing 60–69 kg had a lower risk of revision compared with all other weight groups, whereas weight was not found to affect risk of any revision in patients aged 55–70 years.

Obesity and associated healthcare issues comprise a rising problem worldwide. Several patient-related factors, including obesity, have been reported to be associated with poor outcome following total knee arthroplasty (TKA). Obese TKA patients have an increased risk of infection and deep vein thrombosis (Si et al. 2015, Electricwala et al. 2017), as well as increased load on the prosthesis–bone junction, leading to increased risk of bone or ligament insufficiency and migration (Astephen Wilson et al. 2010). Increased risk for revision following primary TKA has been reported in patients who are overweight (BMI >25) (Morrison 1970, Griffin et al. 1998, Foran et al. 2004a, 2004b, Ward et al. 2015, Zingg et al. 2016). However, patients can have high weight even if BMI is normal. Therefore, it seems justifiable to use weight instead of BMI to assess the stress load on the prosthesis. No previous studies have assessed the relationship between patient weight and revision of the primary TKA in a broad spectrum of TKA patients. Previous studies are limited by small sample size, inclusion of only patients with severe obesity compared with normal BMI, single-center study, and a high number of patients lost to follow-up or a lack of information regarding these patients.

Thus, little is known about whether specific weight indices can be used as cutoffs to determine which patients are at increased risk for having a poor postoperative outcome. We hypothesized that risk of revision of primary TKA increased with increasing weight. Therefore, we conducted a population-based follow-up study to examine the association between weight at the time of primary TKA and risk of any revision as well as risk of revision due to aseptic loosening or infection. 

## Patients and methods

### Study design and settings

We conducted this study in Denmark (5.7 million inhabitants) using prospectively collected data from population-based medical and administrative registers. The Danish National Health Service provides tax-supported healthcare to all Danish residents.

The study was performed in accordance with the Reporting of studies Conducted using Observational Routinely collected health Data (RECORD) statement (Benchimol et al. 2015).

### Data sources

The Danish Knee Arthroplasty Register (DKR) is a national clinical quality database which includes records of all primary TKAs and TKA revisions. The DKR started in January 1, 1997 and collects pre-, peri-, and postoperative data. The DKR is certified by and operates according to requirements of the Danish National Board of Health. Using DKR, we identified our study population, including all patients registered with a primary TKA in DKR in the period January 1, 1997 until end of follow-up on November 19, 2015. From DKR we also obtained information on weight, perioperative complications, type of fixation, indication for primary TKA, and date and indication for revision surgery. The data completeness in DKR was 70% and 56% for primary TKA and revision TKA in 1997, rising to 99% and 96% in 2015 (Knaealloplastikregister 2017).

**Figure 1.**
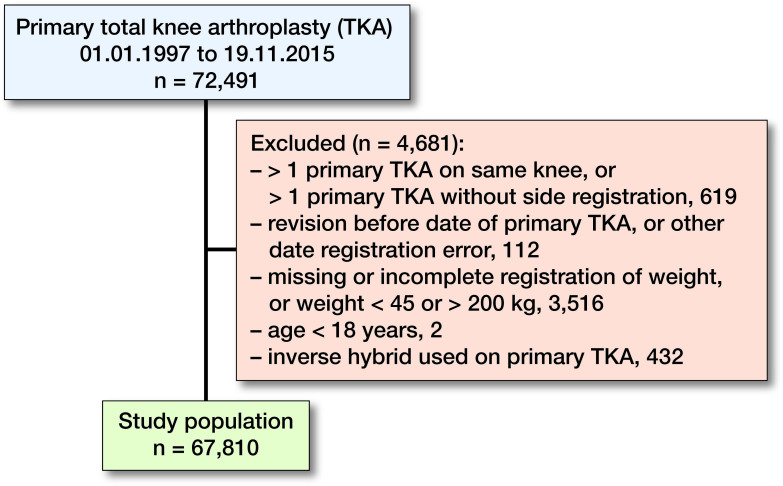
Inclusion and exclusion of patients in the study population. Patients with inverse hybrid fixation techniques were excluded from analyses as this method is rare and the registration is most likely due to error.

Patients registered with more than 1 primary TKA on the same knee, more than 1 primary TKA without information on laterality, revision of TKA prior to primary TKA on the same knee, missing value for weight, weight recorded as either <45 or >200 kg, or registration error of date were excluded from analyses. In addition, the study population was restricted to patients aged at least 18 years of age at the time of primary TKA. Patients with inverse hybrid fixation techniques (cemented femur and cementless tibia) were also excluded from analyses as this method is rare and the registration is most likely due to error (Figure 1). Excluded patients were similar to the included patients with respect to age and sex distribution at the time of primary TKA or indication for primary TKA (data not shown).

Data from DKR were linked to the Danish Civil Registration System (CRS), to obtain information on vital status (active, date of death/emigration). The CRS was established in 1968. All Danish residents (native or immigrated) are registered and assigned a unique personal identification number (CPR number) in the CRS encoding age, sex, and date of birth. The CRS is updated daily. The CPR number allows unambiguous linking between all the Danish medical and administrative registries (Pedersen 2011, Schmidt et al. 2014).

Further, data were linked to the Danish National Patient Register (NPR), which contains nationwide clinical data on inpatients admitted to Danish hospitals since 1977, and outpatients since 1994 (Lynge et al. 2011, Schmidt et al. 2015). Each record in the NPR contains the patient’s CPR number and information on treatment, surgery, dates of admission, primary discharge diagnosis, and up to 20 secondary discharge diagnoses. Diagnoses are classified according to the International Classification of Diseases (ICD). The 8th edition (ICD-8) was used from 1977 to 1993 and the 10th edition (ICD-10) thereafter. We obtained information on complete hospitalization history of each patient from the NPR to assess comorbidity at the time of primary TKA. We computed Charlson’s Comorbidity Index (CCI) at the time of primary TKA and translated into corresponding ICD-8 and ICD-10 codes, similar to the approach by Deyo et al. (1992) (see Supplementary data). We classified patients into 3 levels according to the degree of comorbidity: index low (0 points), corresponding to patients with no previous recorded disease categories implemented in CCI; index medium (1–2 points); and index high (≥ 3 points).

### Exposure

Weight at the time of primary TKA was the exposure in our study and patients were categorized into 1 of the following 6 intervals (45–60, 60–69, 70–79, 80–89, 90–99, and 99–200 kg).

### Outcome

The outcome was time to revision following primary TKA, defined as a new surgical intervention involving partial or complete removal of the implant. We analyzed revision due to (1) all-cause, (2) aseptic loosening, or (3) infection.

### Statistics

Follow-up started on the date of the primary TKA, and ended on the date of the 1st revision, death, emigration, disappearance, or end of follow-up on November 19, 2015, whichever came first.

Cumulative incidence function (CIF) was used to estimate cumulative implant failure probabilities as a result of: (1) all-cause, (2) aseptic loosening, or (3) infection including death or emigration as a competing risk.

A Cox proportional hazards analysis was used to assess the risk of revision by computing cause-specific hazard ratios (HR) for the revision with 2-sided 95% confidence interval (CI). All data were analyzed with and without adjustment for a priori selected variables based on potential confounding effects extracted at the time of primary TKA including sex, age (18–55, 55–70, > 70 years); CCI (low, medium, high); indication for primary TKA (primary arthrosis, secondary arthrosis (defined as arthrosis with a known cause, e.g. sequelae after meniscectomy), fracture (tibia, femur, patella), arthritis, or other); fixation technique (cemented, hybrid, cementless); and perioperative complications (yes, no).

We estimated the HRs for revision comparing different weight intervals with the 70–79 kg weight interval (reference). Since there was an interaction between age and weight, the aHR were calculated separately for each of the 3 age groups. P-values of <0.05 were considered to be statistically significant. All statistical analyses were performed using SAS version 9.4 (SAS Institute Inc, Cary, NC, USA).

### Ethics, funding and potential conflicts of interest

The study was approved by the Danish Data Protection Agency (DDPA): approval number 2012-58-0004. By Danish law, ethical approval and informed consent are not required for entirely register-based studies not involving contact with study participants. Financial support was received from PROCRIN (Program for Clinical Research Infrastructure), a grant of 2 months’ salary for statistical and epidemiological support (PV and EB). No conflicts of interest to declare.

## Results

Among 72,491 patients recorded in the DKR, 67,810 were included in final analyses. Median follow-up time was 5.4 years. A total of 3,270 patients sustained all-cause revision (4.8%), of which 1,109 (34%) were due to aseptic loosening, 173 (5.3%) due to infection and 1,988 (61%) due to other causes including pain without loosening, instability, secondary insertion of patella component, replacement of polyethylene, second part of 2-stage revision, progression of arthrosis and other causes (Table 1). The median age at time of primary TKA was 68 years and men represented 39% of the study population. Fixation of primary TKA was mainly cemented (77%), with hybrid (femur cementless and tibia cemented) and cementless techniques representing 16% and 7.7% respectively. A high comorbidity (CCI >2) was found in 18,099 patients (27%) at the time of their primary TKA (Table 1).

**Table 1.** Demography across weight groups

Parameter Value	No. of patients	Weight (kg)						p-value**^a^**
		45–-60	60–69	70–79	80–89	90–99	99–200	
Subjects ^b^	67,810	3,074 (4.5)	9,336 (13.8)	16,336 (24.1)	16,538 (24.4)	10,585 (15.6)	11,941 (17.6)	
Sex ^b^								
Women	41,506	2,947 (4.3)	8,182 (12.1)	11,492 (16.9)	9,072 (13.4)	4,767 (7.0)	5,046 (7.4)	< 0.001
Men	26,304	127 (0.2)	1,154 (1.7)	4,844 (7.1)	7,466 (11.0)	5,818 (8.6)	6,895 (10.2)	
Age at primary TKA (overall median age: 68) median, years	67,810	74	72	70	68	66	64	< 0.001
Charlson’s Comorbidity Index (CCI) ^c^								
Low (CCI 0)	38,355	1,535 (49.9)	5,255 (56.3)	9,349 (57.2)	9,502 (57.5)	6,090 (57.5)	6,624 (55.5)	
Medium (CCI 1–2)	11,356	471 (15.3)	1,519 (16.3)	2,758 (16.9)	2,786 (16.8)	1,785 (16.9)	2,037 (17.1)	
High (CCI >2)	18,099	1,068 (34.7)	2,562 (27.4)	4,229 (25.9)	4,250 (25.7)	2,710 (25.6)	3,280 (27.5)	
Indication for primary TKA ^c^**^, d^**								
Primary arthrosis	55,683	2,266 (74.2)	7,536 (81.3)	13,599 (84.1)	13,662 (83.4)	8,707 (83.0)	9,913 (83.7)	
Secondary arthrosis	7,220	257 (8.4)	850 (9.2)	1,519 (9.4)	1,867 (11.4)	1,303 (12.4)	1,424 (12.0)	
Sequelae after fracture	1,538	157 (5.1)	267 (2.9)	384 (2.4)	316 (1.9)	199 (1.9)	216 (1.8)	
Arthritis	2,182	337 (11.0)	506 (5.5)	552 (3.4)	413 (2.5)	184 (1.8)	190 (1.6)	
Other ^e^	595	39 (1.3)	108 (1.2)	121 (0.8)	134 (0.8)	97 (0.9)	96 (0.8)	
Fixation technique of primary TKA ^c^								
Cemented		2,465 (80.2)	7,237 (77.5)	12,307 (75.3)	12,766 (77.2)	8,058 (76.1)	9,125 (76.4)	
Uncemented		215 (7.0)	697 (7.5)	1,346 (8.2)	1,275 (7.7)	812 (7.7)	906 (7.6)	
Hybrid		394 (12.8)	1,402 (15.0)	2,683 (16.4)	2,497 (15.1)	1,715 (16.2)	1,910 (16.0)	
Revision TKA (rTKA) ^c^	3,270	136 (4.4)	337 (4.0)	751 (4.6)	807 (4.9)	530 (5.0)	709 (5.9)	
Perioperative complications ^c^	589	35 (1.1)	105 (1.1)	136 (0.8)	130 (0.8)	77 (0.7)	106 (0.9)	
Time to revision (overall median time to revision: 1.88 years) median, years	3,270	1.86	1.94	1.85	1.95	1.89	1.78	0.9
Indication for rTKA ^c^								
Aseptic loosening	1,109	43 (31.6)	98 (29.1)	276 (36.8)	272 (33.7)	177 (33.4)	243 (34.3)	
Infection	173	4 (2.9)	19 (5.6)	37 (4.9)	42 (5.2)	34 (6.4)	37 (5.2)	
Other ^e^	1,988	89 (65.4)	220 (65.3)	438 (58.3)	493 (61.1)	319 (60.2)	429 (60.5)	
Deaths and emigration (overall 18.6%) during follow-up ^c^	12,632	906 (29.5)	2,153 (23.1)	3,407 (20.9)	2,879 (17.4)	1,532 (14.5)	1,755 (14.7)	
Follow-up time (years), median								
Non-revised	64,540	5.4	5.7	5.8	5.6	5.2	5.0	
Revision—all cause	3,270	1.9	1.9	1.9	2.0	1.9	1.8	
Revision—aseptic loosening	1,109	3.6	2.8	3.0	3.0	2.6	2.9	
Revision—infection	173	3.4	1.9	1.6	0.6	1.3	0.8	

**^a^**For categorical variables the chi-squared test was used and for continuous variables the Kruskal–Wallis test was used. P-value <0.05 indicates a significant difference between values in same row.

**^b^**Frequency and % of total

**^c^**Frequency and % of patients in each weight group

**^d^**The total number of included patients is 67,810; due to missing data on indication for primary TKA only 67,218 patients are listed.

**^e^**Other indications for revision TKA include: pain without loosening, instability, secondary insertion of patellar component, replacement of polyethylene, 2nd part of 2-stage revision, progression of arthrosis and other causes.

### Association between weight and revision of primary TKA

**Figure 2.**
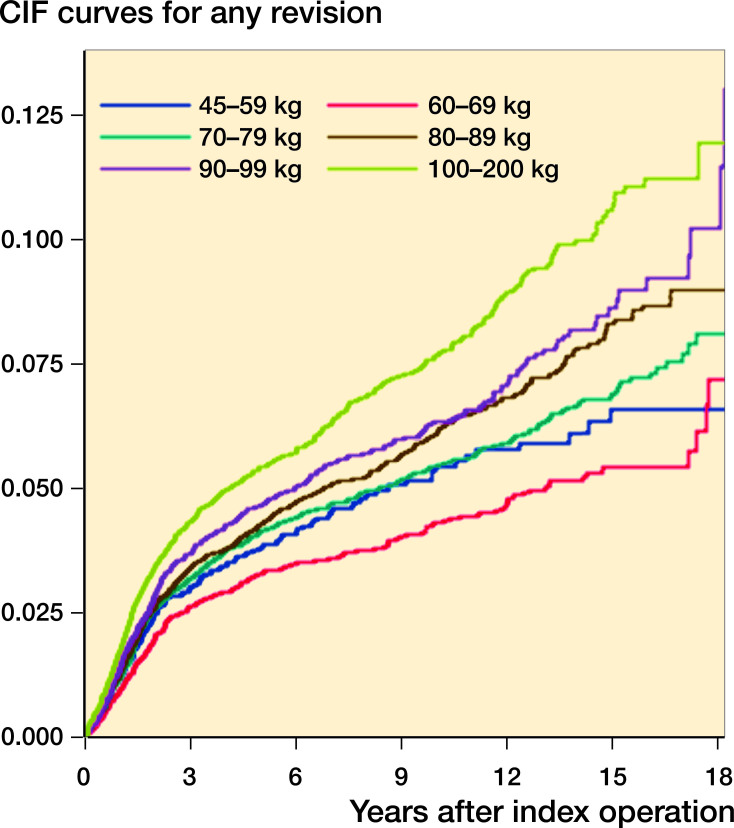
Cumulative all-cause incidence curves for revision of primary TKA according to weight groups.

Patients weighing more than 90 kg had the highest cumulative incidence of all-cause revision during follow-up, whereas patients who weighed 60–69 kg had the lowest cumulative incidence of revision (Figure 2). The cumulative incidences for the different weight groups were shown to be significantly different by utilization of the non-parametric Gray’s test (< 0.001). After 10 years, the CIF were 0.05 (standard error 0.005), 0.04 (0.003), 0.05 (0.002), 0.06 (0.002), 0.06 (0.003), and 0.08 (0.003) for the weight groups 45–60 kg, 60–69 kg, 70–79 kg, 80–89 kg, 90–99 kg, and 99–200 kg, respectively. After approximately 17 years, the accuracy declines due to the low number of patients with such long follow-up (Figure 2).

Crude HRs for all-cause revision were 1.2 (CI 1.1–1.3) and 1.4 (CI 1.3–1.6) for weight groups 90–99 kg and 99–200 kg, and 0.8 (CI 0.7–0.9) for weight group 60–69 kg compared with patients in weight group 70–79 kg (Table 2). In the adjusted analyses in which sex, weight, and age were adjusted for, a statistically significant interaction between age and weight groups was observed, thus these variables could not be considered separately (data not shown). In the youngest age group (18–55 years), patients in the weight group 60–69 kg had a lower risk of revision (HR =0.7, CI 0.5–1.0) compared with the reference group (70–79 kg). Higher weight in the youngest patients was not associated with increased risk of all-cause revision (Table 2). In the age group >70 years, the weight groups 45–60 kg, 80–89 kg, 90–99 kg, and 90–200 kg had a 40–68% increased risk of all-cause revision compared with the weight group 70–79 kg.

**Table 2.** Crude and adjusted hazard ratio (HR) with 95% confidence interval (CI) for revision (all causes) according to different weight and age groups. Values are number of subjects (95% CI)

HR	No. of patients	Weight (kg)						p-value**^a^**
		45–-60	60–69	70–79 (ref.)	80–89	90–99	99–200	
Crude HR	67,810	0.98 (0.82–1.20)	0.79 (0.69–0.89) ^b^	1	1.09 (0.99–1.21)	1.17 (1.05–1.31) ^b^	1.40 (1.27–1.55) ^b^	< 0.001
Adjusted HR ^c^								
18–55 years	7,151	0.91 (0.61–1.36)	0.73 (0.54–0.98) ^b^	1	0.92 (0.74–1.15)	0.89 (0.70–1.13)	0.96 (0.78–1.18)	< 0.001
55–70 years	32,218	0.86 (0.63–1.18)	0.84 (0.70–1.00)	1	0.90 (0.78–1.04)	0.89 (0.76–1.04)	1.07 (0.93–1.23)	
> 70 years	27,849	1.40 (1.05–1.87) ^b^	0.90 (0.71–1.13)	1	1.48 (1.22–1.79) ^b^	1.68 (1.34–2.11) ^b^	1.60 (1.25–2.05) ^b^	

**^a^**P-value for linear trend

**^b^**Significant, p < 0.05

**^c^**Adjusted for sex, comorbidities, perioperative complications, years after primary TKA, type of fixation, and indication for primary TKA.

Due to missing values in data, the total number of patients in the adjusted calculations is 67,218.

### Association between weight and revision due to aseptic loosening

**Figure 3.**
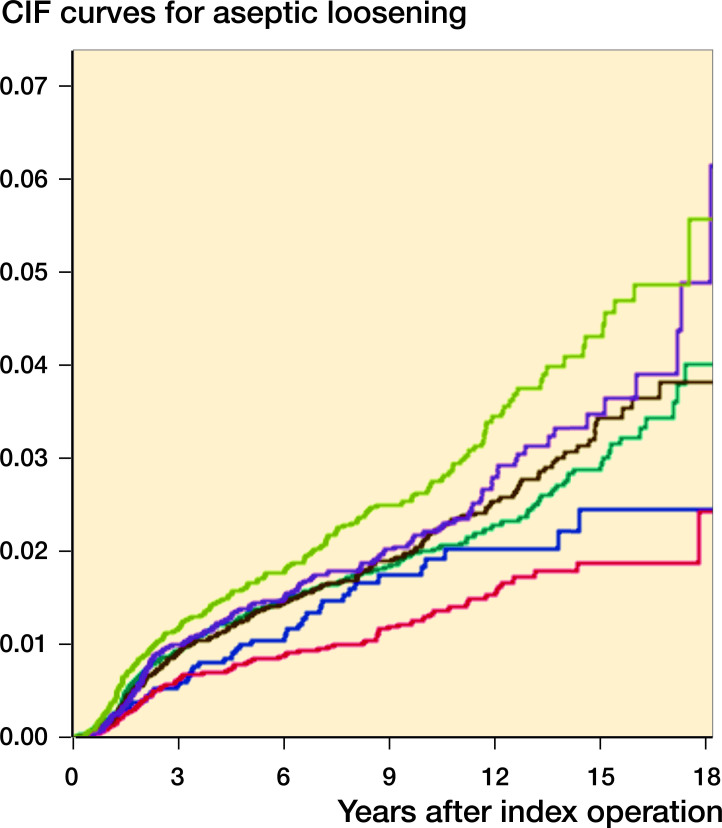
Cumulative incidence curves for revision of primary TKA due to aseptic loosening for weight groups. For color codes, see Figure 2

We found reduced risk of revision due to aseptic loosening (aHR =0.7, CI 0.5–0.8) for the weight group 60–69 kg compared with the group 70–79 kg. There was no association between other weight groups and revision due to aseptic loosening when adjusting for all available confounders (Table 3). The cumulative incidences for the different weight groups were likewise shown to be significantly different by utilization of the non-parametric Gray’s test (< 0.001). After 10 years, the CIF were 0.02 (0.003), 0.01 (0.002), 0.02 (0.001), 0.02 (0.002), 0.02 (0.002), and 0.03 (0.002) for the weight groups 45–60 kg, 60–69 kg, 70–79 kg, 80–89 kg, 90–99 kg, and 99–200 kg, respectively. As for all-cause revision, the accuracy declines after approximately 17 years due to low number of patients (Figure 3).

**Table 3.** Crude and adjusted hazard ratio (HR) with 95% confidence interval (CI) for revision due to aseptic loosening according to different weight and age groups. Values are number of subjects (95% CI)

Parameter	No. of patients	Weight (kg)						p-value**^a^**
		45–-60	60–69	70–79 (ref.)	80–89	90–99	99–200	
Crude HR	67,810	0.85 (0.62–1.18)	0.62 (0.50–0.79) ^b^	1	1.02 (0.86–1.20)	1.09 (0.91–1.32)	1.35 (1.13–1.60) ^b^	< 0.001
Adjusted HR**^c^**	67,218	0.91 (0.65–1.26)	0.66 (0.52–0.83) ^b^	1	0.97 (0.82–1.15)	0.96 (0.79–1.17)	1.09 (0.91–1.30)	0.004

**^a^**P-value for linear trend

**^b^**Significant, p < 0.05

**^c^**Adjusted for sex, age, comorbidities, perioperative complications, years after primary TKA, type of fixation, and indication for primary TKA.

Due to missing values in data, the total number of patients in the adjusted calculations is 67,218.

### Association between weight and revision due to infection

The cumulative incidences for the risk of revision due to infection between weight groups were not statistically significantly different (data not shown). However, it should be noted that few patients were revised due to infection (173), with only 4 patients in the lowest weight group.

Revision due to infection was not found to be related to weight groups (Table 4).

**Table 4.** Crude and adjusted hazard ratio (HR) with 95% confidence interval (CI) for revision due to infection according to different weight and age groups. Values are number of subjects (95% CI)

Parameter	No. of patients	Weight (kg)						p-value**^a^**
		45–-60	60–69	70–79 (ref.)	80–89	90–99	99–200	
Crude HR	67,810	0.58 (0.21–1.64)	0.90 (0.52–1.56)	1	1.14 (0.74–1.78)	1.49 (0.93–2.37)	1.45 (0.92–2.29)	0.2
Adjusted HR**^b^**	67,218	0.71 (0.25–2.02)	0.98 (0.55–1.73)	1	0.98 (0.62–1.53)	1.08 (0.67–1.75)	1.00 (0.62–1.62)	1.0

**^a^**P-value for linear trend

**^b^**Adjusted for sex, age, comorbidities, perioperative complications, years after primary TKA, type of fixation, and indication for primary TKA.

Due to missing values in data, the total number of patients in the adjusted calculations is 67,218.

## Discussion

In this nationwide cohort study, we assessed the effects of weight on the risk of revision following primary TKA. We found an association between weight above 80 kg and increased risk of all-cause revision in patients aged over 70 years at the time of primary TKA. Further, patients over 70 years who weighed 45–60 kg were at increased risk of all-cause revision compared with patients weighing 70–79 kg. There was no indication of dose response. Our findings imply that high weight for aged patients is an important risk factor for revision of primary TKA. In the crude analysis and in the cumulative all-cause incidence we found that patients weighing 60–69 kg had the lowest risk of revision.

Whether obesity is associated with higher revision rate is debatable. Bordini et al. (2009) found no different rates of survival in obese patients compared with normal-weight patients at 5 year follow- up. However, the total number of TKAs in that study was only 9,735 with 186 revisions and a revision rate of 1.9%. The number of morbidly obese patients having revision was only 4 and only 59 obese patients had revision; it is therefore not unlikely that no difference were found as some subgroups had a low number of patients included. Several researchers suggest, however, that obesity is associated with increased risk of revision (Si et al. 2015, Ward et al. 2015, Werner et al. 2015, Zingg et al. 2016, Electricwala et al. 2017).

Obesity is a risk factor for primary arthrosis and primary arthrosis is the main indication for primary TKA; it is therefore expected to find lower age among the heaviest patients. Shah et al. (2017) found a significantly higher occurrence of obesity and morbid obesity among patients <65.We could not show that increased weight was associated with increased risk of revision in younger patients, but patients weighing 60–69 kg had a lower risk of revision. These results may be due to the lower number of patients aged 18–55 years. We cannot explain why patients >70 years with high weight are at higher risk of revision than younger patients of the same weight.

Revisions due to aseptic loosening represented 34% of the revisions and were thereby the single largest indication for revision TKA among our patients. The CIF curves show, similar to crude HR, that only patients weighing 60–69 kg and 99–200 kg were at statistically significantly different risk than the reference group. Even though aseptic loosening is the commonest indication for revision, it still only represents 1,109 patients, which might not be enough to show a statistically significant difference between weight groups. Electricwala et al. (2017) found aseptic loosening to be the 3rd highest reason for revision after infection and instability. They also found that not only obese patients but also overweight patients had a higher risk of revision. In other studies, Zingg et al. (2016) and Ward et al. (2015) similarly found a higher risk of revision with rising BMI, but did not separate their results into reasons for revision.

Revisions due to infection disclosed a completely different pattern in which no statistically significant difference in risk of revision for different weight groups was observed, which backs the notion that infection occurs randomly across risk groups. This might be due to the low occurrence of infection leading to revision; only 173 patients were revised due to infection or 0.26% of patients.

TKA is a safe procedure and the overall revision rate in our data was 4.8% with a median follow-up time of 5.4 years. Obesity is correlated with a number of risk factors and comorbidities affecting major surgery like TKA. It is strongly advisable for overweight patients to lose weight prior to TKA surgery. Both our results regarding stress load on the prosthesis in relation to patient weight and other comparable studies show increased risk of revision in overweight patients. Among our patients older than 70 years the revision risk was statistically increased even when adjusted for a number of a priori chosen confounders.

### Strengths and limitations

The completeness of DKR is high, especially since 2007, but some patients have been lost during registration. A validation of the Danish Hip Arthroplasty Register regarding reporting of infections to the register has been published. Several data sources including NPR were used, and showed an estimated “true” incidence of surgically treated infections to be 40% higher than reported by national arthroplasty registries alone (Gundtoft et al. 2015). To our knowledge no similar study has been undertaken with data from the DKR, but we may expect some degree of under-reporting of infections to the DKR as well.

When assessing our results, we adjusted for a number of a priori chosen confounders such as sex, age, comorbidities, peroperative complications, years after primary surgery, type of fixation, and indication for primary TKA. But residual confounders may occur because, for example, we lack data on comorbidities recorded at general practitioners, and on psychiatric comorbidities, as well as severity of some comorbidities included in the CCI.

Other risk factors like alcohol and smoking were not available in our dataset, causing unmeasured confounding. Likewise it was not possible to investigate the correlation between BMI and revision rate of primary TKA, since registration of patient height first began nationwide in 2011. Most studies use BMI to graduate obesity, but with disadvantages, since this does not account for the absolute weight load on the prosthesis. Percentage body fat has been shown to be superior to BMI but those data were not available in our registries (Ledford et al. 2014).

### Summary and perspectives

We found an increased risk of any revision following primary TKA in patients older than 70 years of age weighing <60 kg and >80 kg. Patients aged 18–55 years weighing 60–69 kg had a lower risk of revision compared with all other weight groups, whereas weight did not affect risk of any revision in patients 55–70 years.

### Data sharing

If approved by the DDPA access to raw data will be granted on request. Access to protocol and programming code will be granted on request by contact with the corresponding author.

### Supplementary data

Charlson Comorbidity Index (CCI) and Tables 3 and 4 are available as supplementary data in the online version of this article, http://dx.doi.org/10.1080/17453674.2018.1540091

All authors contributed significantly to this paper. DG, KG, and AT jointly initiated the study and wrote the protocol. EB modified the protocol and applied for approval by the DDPA. DG wrote the main paper. PV performed the statistics and wrote the statistics rapport. All authors performed critical revision of the paper.

*Acta* thanks Martin Lindberg-Larsen and Maziar Mohaddes for help with peer review of this study.

## Supplementary Material

Supplemental Material

## Charlson Comorbidity Index (CCI)

1 point Myocardial infarction

Congestive heart failure

Peripheral vascular disease

Cerebrovascular disease

Dementia

Chronic pulmonary disease

Connective tissue disease

Ulcer disease

Mild liver diseas

Diabetes I and II

2 points Hemiplegia

Moderate to severe renal disease

Diabetes with end organ damage

Any tumor

Leukemia

Lymphoma

3 points Moderate to severe liver disease

6 points Metastatic solid tumor

AIDS

CCI is the sum of points. We classified patients into 3 levels according to the degree of comorbidity: index low (0 points), corresponding to patients with no previous recorded disease categories implemented in CCI; index medium (1–2 points); and index high (≥ 3 points).
