# Supplementary material for: Weight affects survival of primary total knee arthroplasty: study based on the Danish Knee Arthroplasty Register with 67,810 patients and a median follow-up time of 5 years
Source: Acta Orthop. 2018 Dec 5;90(1):60–6. doi: 10.1080/17453674.2018.1540091 (PMC6366474; doi:10.1080/17453674.2018.1540091)
Supplement: Supplemental Material [file IORT_A_1540091_SM0870.pdf]

Supplementary data

Charlson Comorbidity Index (CCI)

1 point	Myocardial infarction<br>Congestive heart failure<br>Peripheral vascular disease<br>Cerebrovascular disease<br>Dementia<br>Chronic pulmonary disease<br>Connective tissue disease<br>Ulcer disease<br>Mild liver diseas<br>Diabetes I and II
2 points	Hemiplegia<br>Moderate to severe renal disease<br>Diabetes with end organ damage<br>Any tumor<br>Leukemia<br>Lymphoma
3 points	Moderate to severe liver disease
6 points	Metastatic solid tumor<br>AIDS

CCI is the sum of points. We classified patients into 3 levels according to the degree of comorbidity: index low (0 points), corresponding to patients with no previous recorded disease categories implemented in CCI; index medium (1–2 points); and index high (≥ 3 points).

Table 3. Crude and adjusted hazard ratio (HR) with 95% confidence interval (CI) for revision due to aseptic loosening according to different weight and age groups. Values are number of subjects (95% CI)

Parameter	No. of patients	Weight (kg) 45–-60	60–69	70–79 (ref.)	80–89	90–99	99–200	p-value [a]
Crude HR	67,810	0.85 (0.62–1.18)	0.62 (0.50–0.79) [b]	1	1.02 (0.86–1.20)	1.09 (0.91–1.32)	1.35 (1.13–1.60) [b]	< 0.001
Adjusted HR [c]	67,218	0.91 (0.65–1.26)	0.66 (0.52–0.83) [b]	1	0.97 (0.82–1.15)	0.96 (0.79–1.17)	1.09 (0.91–1.30)	0.004

[a] P-value for linear trend
[b] Significant, $p < 0.05$
[c] Adjusted for sex, age, comorbidities, perioperative complications, years after primary TKA, type of fixation, and indication for primary TKA. Due to missing values in data, the total number of patients in the adjusted calculations is 67,218.

Table 4. Crude and adjusted hazard ratio (HR) with 95% confidence interval (CI) for revision due to infection according to different weight and age groups. Values are number of subjects (95% CI)

Parameter	No. of patients	Weight (kg) 45–-60	60–69	70–79 (ref.)	80–89	90–99	99–200	p-value [a]
Crude HR	67,810	0.58 (0.21–1.64)	0.90 (0.52–1.56)	1	1.14 (0.74–1.78)	1.49 (0.93–2.37)	1.45 (0.92–2.29)	0.2
Adjusted HR [b]	67,218	0.71 (0.25–2.02)	0.98 (0.55–1.73)	1	0.98 (0.62–1.53)	1.08 (0.67–1.75)	1.00 (0.62–1.62)	1.0

[a] P-value for linear trend
[b] Adjusted for sex, age, comorbidities, perioperative complications, years after primary TKA, type of fixation, and indication for primary TKA. Due to missing values in data, the total number of patients in the adjusted calculations is 67,218.
